# Modulating auditory attention and stimulus evaluation in the medial prefrontal cortex: A translational tone approach oddball paradigm in rats

**DOI:** 10.3758/s13415-026-01473-7

**Published:** 2026-07-07

**Authors:** Marcel R. Oelerich, Yannis Pfleger, Mesbah Alam, Joachim K. Krauss, Kerstin Schwabe, Franziska M. Decker

**Affiliations:** 1 Department of Neurosurgery, Hannover Medical School, Carl-Neuberg-Straße 1, 30625 Hannover, Germany; 2 Center for Systems Neuroscience Hannover (ZSN), Bünteweg 2, 30559 Hannover, Germany; 3 Cluster of Excellence, EXC 2177/1 “Hearing4all” of the German Research Foundation, 30625 Hannover, Germany

**Keywords:** Three-class oddball paradigm, Event-related potential, Frequency discrimination, Medial prefrontal cortex, Rats, Attention and cognitive processing

## Abstract

The three-class auditory oddball paradigm is used to study sensory processing, attention, and decision-making. In this paradigm, a rare behaviorally relevant target is presented among frequent behaviorally irrelevant standard and rare distractor tones. The Tone Approach Paradigm (TAP) was designed as a variation of this paradigm in which task difficulty increases as the target and distractor frequencies sequentially approach the frequency of the standard tone to investigate neurocognitive aspects of processing behaviorally relevant auditory stimuli under challenging hearing conditions in rats. Male Sprague-Dawley rats (*n* = 10) were trained to respond to target (5000 Hz), while ignoring distractor (1500 Hz) and standard tones (3000 Hz) in baseline (BSL) condition. After training, electrodes were implanted in the medial prefrontal cortex (mPFC) for local field potential recording during behavioral testing. Starting from BSL, the frequency differences between target and distractor to standard tones in the TAP sessions were reduced by 500 Hz per session. Hit ratios for targets declined with increasing difficulty of TAP sessions (*p* < 0.05). Cumulative distributions of reaction times shifted from a sigmoidal profile with stimulus-locked behavior to a more linear profile, suggesting less temporally clustered responding. Event-related potential amplitudes increased at low and moderate TAP difficulty, indicating higher processing effort, but decreased at the highest difficulty, suggesting impaired processing. Our findings show that manipulating task difficulty via frequency changes directly affects behavior and neural processing within the mPFC. TAP provides a translational framework for investigating neurophysiological mechanisms of attention and stimulus evaluation, with relevance for models of attention-related dysfunction.

**Supplementary Information:**

The online version contains supplementary material available at https://doi.org/10.3758/s13415-026-01473-7.

## Introduction

The ability to distinguish relevant from irrelevant information, especially in challenging hearing scenarios, is essential for goal-directed behavior in daily life but it is often disturbed in neuropsychiatric disorders. The three-class auditory oddball paradigm enables the study of neurocognitive mechanisms involved in distinguishing behaviorally relevant auditory stimuli from irrelevant ones. It is used to investigate the neural resources supporting attention during stimulus processing under challenging listening conditions. In the oddball paradigm, subjects are presented with standard stimuli that occur frequently and are to be ignored. These are occasionally interrupted by rare deviant stimuli, which either require a behavioral response as targets or must be ignored as distractors (Debener et al., 2002; Decker et al., 2025a). We recently showed that event-related potentials (ERPs) recorded from the medial prefrontal cortex (mPFC) of rats during behavioral testing in the three-class oddball paradigm share several features with human ERPs, highlighting the translational relevance of this model (Decker et al., 2025a; Shinba, 1997).

In human research, frequency, intensity and other acoustic stimulus features in oddball paradigms have been manipulated to create a different levels of discrimination difficulty (Combs & Polich, 2006; Katayama & Polich, 1998; Polich, 1987; Sanada et al., 2025). In human electroencephalography (EEG) studies, changes in the ERP P3 component have been linked to cognitive load and the ability to discriminate between stimuli (Kok, 2001). Increasing task difficulty in human oddball paradigms has been associated with changes in P3 latency and amplitude. However, once attentional resources are overextended or stimulus discrimination becomes infeasible, the late ERP amplitude may decline (Isreal et al., 1980; Polich, 1987, 2007). The neural mechanisms underlying this complex interplay between behavior and the neural processing of auditory stimuli under these conditions remain incompletely understood.

To gain more direct insight into these neural mechanisms, we introduced the rodent “Tone Approach Paradigm” (TAP) which gradually increases difficulty of the three-class oddball paradigm by sequentially shifting the target and distractor frequencies towards the standard tone. A similar auditory oddball paradigm in freely moving rats has already addressed a related question (Quintela-Vega et al., 2023). In that study, rats were trained to discriminate behaviorally between deviant tones in several variations of the classic oddball paradigm, in which the frequency contrast between standard and deviant tones was systematically varied across a wide range. Our study builds on this behavioral framework by systematically reducing the frequency contrasts between target and standard as well as between distractor and standard in the three-class oddball paradigm, while simultaneously recording electrophysiological activity from the mPFC during behavioral testing. This gradual frequency manipulation resembles human studies in which frequency discrimination is systematically varied (easy and hard conditions) to assess adaptive changes in attention and perceptual decision-making (Novak et al., 1992; Sanada et al., 2025).

Rats were first trained in the three-class oddball paradigm to respond to a 5000 Hz stimulus designated as the target, while ignoring a 3000 Hz stimulus presented as the standard and a 1500 Hz stimulus serving as the distractor. To elucidate the complex interplay between behavioral responses to auditory stimuli and ERP characteristics, rats were then tested under TAP conditions in which the target and distractor frequencies were sequentially shifted toward the standard frequency, thus reducing their discriminability. For behavioral measures, the ratio of correct responses (hits) to the target, respectively correct rejection (CR) of distractors were analyzed. Furthermore, cumulative reaction-time distributions were used as a descriptive measure of stimulus-locked responses to target tones and false alarms (FA) to distractor tones. Previous work by Casado-Román et al. (2020) showed that the mPFC contributes to context dependent processing of deviant auditory stimuli. We therefore analyzed ERPs derived from local field potentials (LFPs) recorded in the mPFC during behavioral testing to investigate the neural processes underlying stimulus evaluation and discrimination during the systematic manipulation of target and distractor frequencies.

## Methods

Adult male Sprague Dawley rats (*n* = 10) were housed in groups of two to four rats in Makrolon Type IV cages in a scantainer (Scanbur, Karlslunde, Denmark; 22 ± 2 °C; 55 ± 10% humidity; 10/14 hr dark/light cycle, lights on at 06:00 a.m.) with free access to water. Standard rodent chow (Altromin, Lage, Germany) was provided with 16 g per day per animal to enhance the salience of reward pellets during behavioral testing while still ensuring weight gain to a body weight (BW) of approximately 400 g. Clinical scores and BW were assessed at least twice a week to monitor the animals’ well-being, in addition to daily visual inspections. All procedures were approved by the local authority LAVES (Niedersächsisches Landesamt für Verbraucherschutz und Lebensmittelsicherheit, AZ 23–00312) and were conducted in accordance with EU Directive 2010/63/EU.

### Training the oddball paradigm

Rats were trained in behavioral chambers (80600A Operant Chamber, Campden Instruments Limited, Loughborough, UK) equipped with one open hole in the back panel and a small container with a flap on the front panel for reward pellets (Dustless Precision Pellets 45 mg, Rodent Purified Diet, BioServ) delivered via a food magazine. The hole in the back contained an infrared light beam that detected nose-poke responses following stimulus presentation. An additional infrared sensor in the front container detected reward pellet retrieval after a correct hit. The stimulus schedules and the data acquisition were controlled by Presentation software (Neurobehavioral Systems Inc., Berkeley, CA), which also sent signals to a microcontroller (Arduino) that controlled the feeder's stepper motor. Auditory stimuli were presented via loudspeakers and were audible throughout the entire chamber. At all times, rats were allowed to move freely within the chamber.

**Fig. 1:**
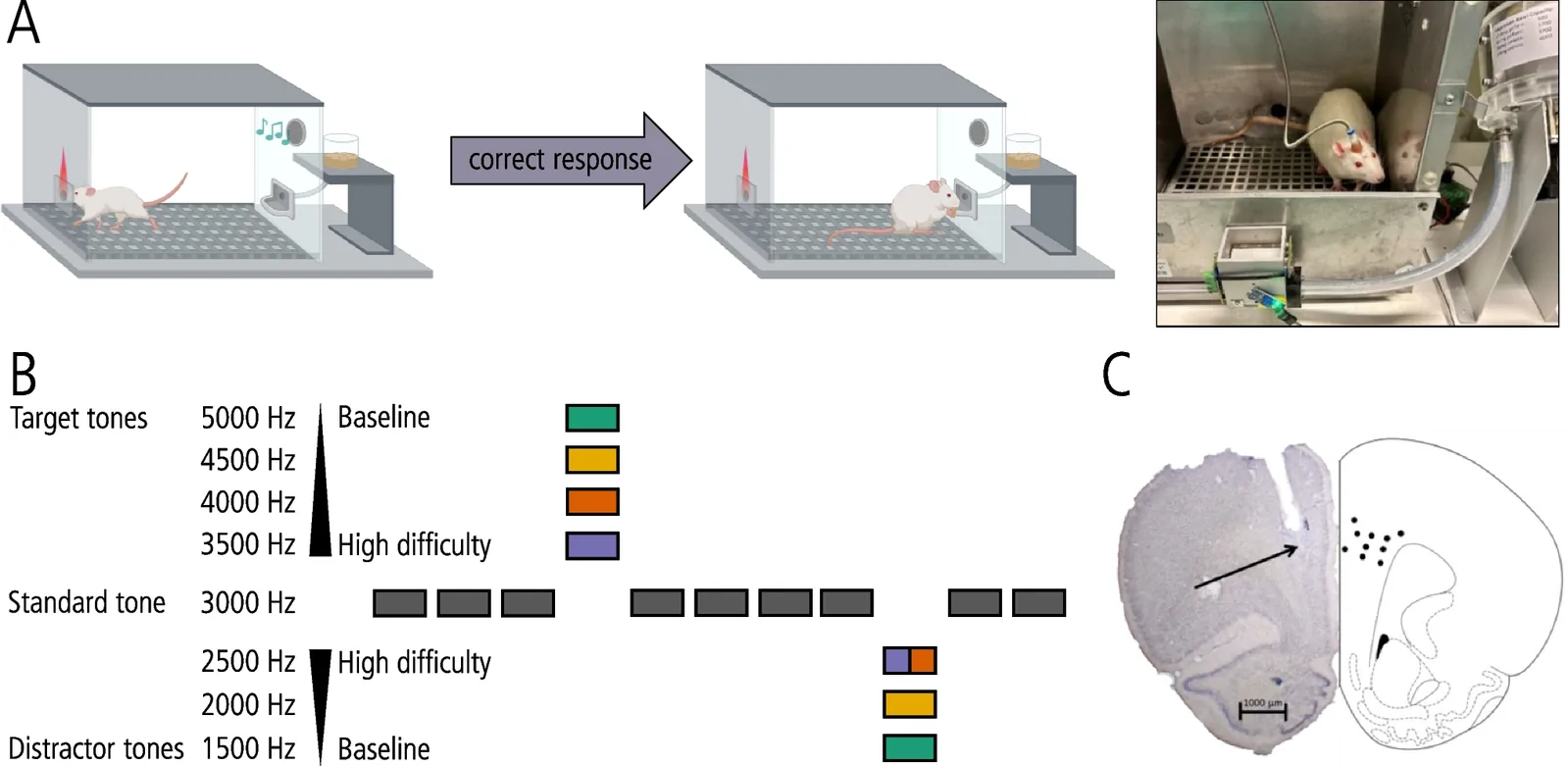
Experimental setup: Behavioral setup in the oddball chambers, showing one open hole located on the back panel for poke detection and a small container in the front panel for reward pellet delivery, accompanied by a photograph for illustration (**A**). Schematic overview of the Tone Approach Paradigm (TAP) across all stages, illustrating target, distractor, and standard frequencies (**B**). Coronal brain section from histological preparation, showing postmortem verification of electrode placement in the medial prefrontal cortex (**C**); (partially created with BioRender.com)

During the three-class oddball paradigm, rats were required to respond by nose-poke in the back hole to a behaviorally relevant target tone (5000 Hz) to obtain a casein pellet and to withhold their responses to a frequent standard tone (3000 Hz) and a rare distractor tone (1500 Hz; Fig. 1A). Training proceeded in several phases with gradually increasing difficulty. First, only target tones were presented until the animals responded correctly on at least 80% of trials. In the next phase, the standard tone was introduced, and rats had to withhold responses to the standard tone on at least 80% of trials while maintaining at least 80% correct responses to the target tone. Once this criterion was reached, the rare distractor tone was added, and rats had to actively learn to ignore distractor tones on at least 80% of trials. Across phases, task difficulty was increased by shortening the trial duration and tone length and by adjusting the time-out penalty after incorrect responses to standard and distractor tones (for more detailed training procedure, see Decker et al., 2025a).

In the final baseline (BSL) oddball sessions, stimuli were presented in a pseudo-random order (standard: 60%, target: 20%, distractor: 20%), and rats were required to respond to target tones by nose poke while correctly ignoring standard and distractor tones on at least 80% of trials. All tones had a duration time of 100 ms, including 5 ms rise and fall times, and were presented with randomly selected inter-trial intervals (ITI) between 1500 and 2500 ms. Each trial started with the presentation of a single tone and was followed by a 3-s response window during which no further stimuli were presented. The response window either ended as soon as the animal responded to the stimulus or after 3 s if no response was made. After a correct nose-poke response to a target stimulus in the back hole, i.e., a hit, rats had to move to the front of the chamber to retrieve the reward pellet from the food magazine. Following a FA to a standard or distractor stimulus, rats were punished with a 1000 ms timeout, in addition to moving to the back hole in vain. The ITI was defined as the delay between the end of the response window (or reward delivery) and the onset of the next tone. Once the rats had reached criterion of 80% correct performance, electrodes were implanted into the mPFC.

### Electrode implantation surgery

Monopolar electrodes (platinum-iridium wire, coated with Teflon Ø 140 µm, uncoated tip of 0.45–0.5 mm, Ø 76.2 µm; Science-Products GmbH) were placed in a 13-mm-long stainless-steel tube cut from a 24-gauge syringe needle (Sterican, Braun Melsungen AG, Germany). The electrodes were stereotaxically implanted into the mPFC under multimodal anesthesia, which included general anesthesia with isoflurane (4% for induction, 2% for maintenance, CP- pharma, Burgdorf, Germany), local anesthetic infiltration along the incision line (2% Xylocaine, AstraZeneca GmbH, Wedel, Germany), and systemic analgesia (Carprofen, 5 mg/kg, Rimadyl, Zoetis, Berlin, Germany). After rats had been secured in a stereotaxic frame, electrodes were implanted in the mPFC with the following coordinates (relative to Bregma, toothbar −3.3 mm): 2.7 mm anterior, ± 0.8 mm lateral and 3.5 mm ventral to the dura, targeting the prelimbic region of the mPFC according to the rat brain atlas (Paxinos & Watson, 2007). This region was selected, because it has been used in previous work on prefrontal–auditory discrimination (Concina et al., 2018; Hockley & Malmierca, 2024).

Reference and grounding electrodes (screws with attached steel wire) were positioned near the recording site and above the cerebellum. A headstage was built with dental acrylic (Paladur, Hanau, Germany) for later connection of a recording cable during behavioral testing. Carprofen analgesia was continued for two postoperative days (2.5 mg/kg), and antibiotics were given for eight days, starting two days prior to surgery (Marbofloxacin, 10 mg in 1 mL/kg, Ismaning, Germany). After 1 week of recovery, the animals resumed training. Electrophysiological recording during behavioral testing began once the animals had regained their preoperative performance level.

### Behavioral and electrophysiological data acquisition during the TAP variant of the oddball paradigm

The TAP comprised four difficulty levels, with the target and distractor frequencies shifting in 500 Hz steps toward the standard tone (Fig. 1B). The three-class oddball paradigm parameters used during training served as the BSL condition, followed by three TAP conditions (TAP1 = low, TAP2 = moderate and TAP3 = high difficulty). The frequency for the standard was consistently set at 3000 Hz. Each TAP session consisted of 200 tone presentations resulting in 40 target and 40 distractor tones per condition. The target, standard, and distractor frequencies for each stage of the TAP are summarized in Fig. 1B.

Correct target hits within the 3-s response time window in all sessions triggered a reward, while responses to distractors were recorded as FA. Furthermore, the reaction times of all pokes (i.e., hits and FA) occurring in the response window were recorded and analyzed by using cumulative frequency distributions with 200-ms time interval bins. We also calculated the interquartile range (IQR) of the reaction times as a robust measure of precision and response-time variability. Cumulative distributions that rise steeply around a specific time point indicate temporally clustered responding and correspond to a small IQR. In contrast, a flatter and more near-linear increase indicates that responses are more broadly distributed across the analysis window and correspond to a wider IQR. To provide a benchmark for chance-level responding, we therefore included an additional “random control” condition that simulated random nose-poking behavior with a uniform distribution of response times. For each of the ten animals, a Python (version 3.11) script generated random time points between 0 and 3000 ms. The number of time points was based on the average number of nose pokes across the TAP conditions, thereby providing a realistic benchmark.

Neural signals from the mPFC electrodes were transmitted via a cable to an electrode adapter box. A swivel was inserted between the cable and the recording system to prevent cable twisting and to allow free movement during behavioral testing in the operant chamber. LFP signals were amplified (×1000), galvanically isolated, and band-pass filtered (0.5–100 Hz) using an analog signal conditioner (Cambridge Electronic Design, UK). The signals were then digitized (Cambridge Electronic Design CED 1401 Mark II) with a sampling rate of 1000 Hz, and saved for further computations (Spike2, version 6). A 50-Hz notch filter was applied to remove electrical line noise from the digitized signals. Neural signals were synchronized with the auditory stimuli of the oddball paradigm via trigger events.

ERP data were extracted from Spike2 into Python and filtered offline with a fourth order Butterworth band-pass filter (0.5–30 Hz), and epoched from −200 to 800 ms relative to stimulus onset. Baseline correction was applied by subtracting the mean voltage of the prestimulus interval (−200 to 0 ms) from each time point within the respective trial. Trials were excluded when their baseline activity in the prestimulus interval exceeded ±3 standard deviations of the distribution of baseline values across all trials from the respective animal. Only a small proportion of trials was excluded during filtering across all conditions, with overall rejection rates remaining below 5% for both target and distractor trials. After artifact rejection, the remaining trials were z-score normalized by subtracting the mean and dividing by the standard deviation of the signal. These normalized data were then used to compute grand averages. Peak amplitudes of the early and late ERP components were then determined for target, distractor, and standard trials. Responses to standard tones were included as reference conditions, with standard FAs serving as the basis for target-related ERP analyses and standard CRs serving as the basis for distractor-related ERP analyses. To assess whether the late ERP component might reflect a filtering artifact, grand-average waveforms were additionally inspected without offline filtering. In addition, the temporal response of the acquisition hardware high-pass filter (0.5 Hz cutoff; time constant 318 ms) was evaluated in relation to the latency and morphology of the observed late ERP component. After completion of behavioral testing, all rats were intracardially perfused with a formalin solution. Thereafter, brains were histologically processed to verify correct electrode placement within the prelimbic subregion of the mPFC (Fig. 1C).

### Statistical analyses

Statistical analyses were conducted in SigmaPlot (version 15.0), GraphPad Prism (version 10), and Python (version 3.11). Behavioral performance for target hits and distractor CRs, expressed as percentages, during the TAP sessions was evaluated using one-way repeated-measures (RM) ANOVA with Tukey’s post hoc tests. Furthermore, signal detection measures (d’-prime (d’) and criterion) were calculated across the TAP conditions and analyzed using a one-way RM ANOVA followed by Bonferroni-corrected post hoc *t*-tests. The Frequency distribution of the reaction times was analyzed using a two-way RM ANOVA (1^st^ Factor: TAP sessions, 2^nd^ Factor: 200 ms time interval bins). For the IQR of the reaction times, a one-way RM ANOVA with Tukey’s post hoc test was used. Target hit and target miss ERPs were also plotted separately for each TAP condition. Grand-average ERPs were compared using permutation-based ANOVA. Statistical significance was assessed using 2000 permutations. Significant effects were followed by pairwise comparisons, with *p*-values corrected for multiple testing using the Benjamini-Hochberg false discovery rate (FDR) procedure. Effect sizes were reported as pooled Cohen’s d. Peak amplitudes of the early and late ERP components were assessed using a one-way ANOVA with Tukey’s post hoc test. The significance threshold was set at *p* < 0.05 (two-sided). Figures were prepared in GraphPad Prism, and ERP plots were generated in Python using Matplotlib (version 3.7.1). Data are presented as mean + SEM.

## Results

**Fig. 2:**
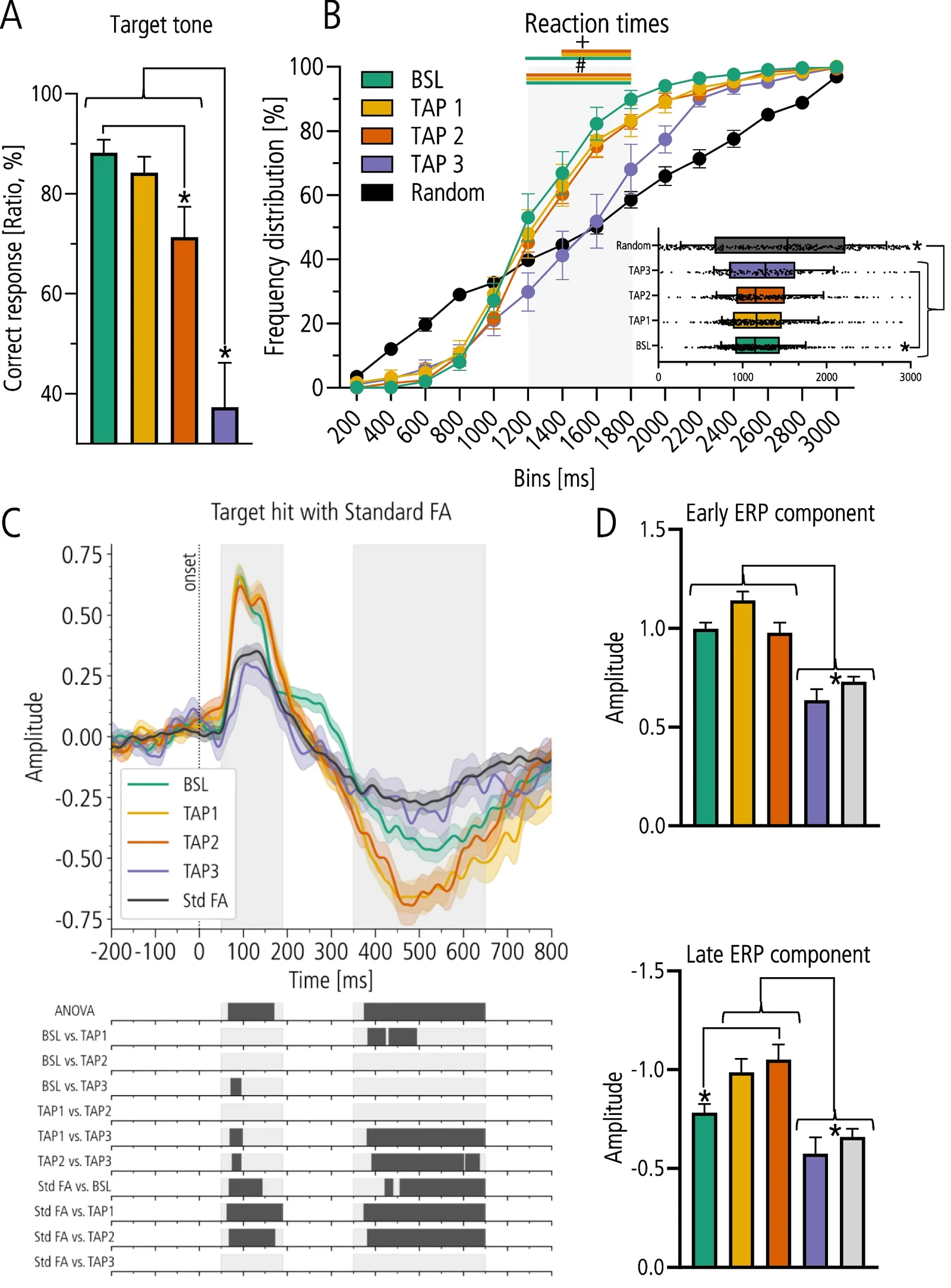
Behavioral and electrophysiology results of the target hit event. Green BSL, yellow TAP1, orange TAP2, purple TAP3. Significant differences (*p* < 0.05) are visualized by asterisk signs (*). Ratio of correct hits to the target tone. Data are shown as mean + SEM (**A**). Frequency distribution of reaction times for all experimental conditions and the simulated random control condition, as well as their associated box plots. Horizontal bars above the curves denote, by color, significant differences between conditions. Hashtag sign (#) against TAP3 and plus (+) against the random control condition (**B**). Grand-averaged ERPs (mean ± SEM) for all conditions aligned to stimulus onset. Gray shaded bands indicate the predefined analysis windows for the early (50–190 ms) and late (350–650 ms) component. Permutation ANOVA shows time segments with significant effects and post hoc comparison (black bars, **C**). Quantitative ERP amplitudes for target hits and standard FA are shown as bar plots summarizing early (top) and late (bottom) amplitudes (mean + SEM), significant differences are visualized by asterisk signs (*p < 0.05; **D**). *BSL* baseline; *ERP* event-related potential; *FA* false alarm; *Std* standard; *SEM* standard error of the mean; *TAP* Tone Approach Paradigm

All animals recovered from surgery within one week and were subjected to neural recording. All electrodes were placed within the prelimbic subregion of the mPFC, and data from all animals were included in the analysis (Fig. 1C). Correct responses to target tones declined with increasing difficulty across the TAP conditions (Fig. 2A). We first report the behavioral results, including accuracy, d’, criterion, and reaction time, before turning to the electrophysiological findings.

A one-way RM ANOVA revealed a significant effect of the factor TAP condition on the correct hit ratio (F_3,27_ = 20.86, *p* < 0.001). Post hoc comparisons indicated that while the correct hit ratio in TAP1 did not differ from that of BSL, performance in all other TAP conditions (TAP2 and TAP3) was significantly reduced (*p* < 0.05). Moreover, during the TAP3 condition, the correct hit ratio was significantly reduced compared with TAP1 and TAP2 (*p* < 0.05). Across conditions, d’ for target discrimination closely followed target hit performance. False alarm rates to distractor and standard tones remained largely stable at around 20% across TAP sessions. Accordingly, d’ was preserved in BSL, TAP1, and TAP2, but was clearly reduced in TAP3, indicating impaired discrimination between target and non-target tones under the highest difficulty condition in both comparisons (F_3,26_ = 8.987 and F_3,26_ = 19.326, *p* < 0.001). A different but complementary pattern was observed for the criterion. One-way ANOVA showed significant differences between TAP conditions (F_3,26_ = 9.792 and F_3,26_ = 12.896, *p* < 0.001). BSL and TAP1 values were close to zero. In TAP2, the criterion was slightly positive, but did not differ significantly from BSL and TAP1 (all *p* > 0.05). Only in TAP3 did criterion differ significantly from all other conditions (*p* ≤ 0.007; Supplementary Fig. S1).

In the BSL condition the cumulative reaction-time distribution had a sigmoidal shape. This sigmoidal distribution differed significantly from those observed in TAP3 and in the simulated random control condition, both of which showed flatter, more linear cumulative profiles. Analysis of the frequency distribution of reaction times with a two-way RM ANOVA showed a significant interaction between the TAP conditions (including the random control condition) and the time interval (F_56,504_ = 10.231, *p* < 0.001). Post hoc comparisons confirmed that the sigmoidal distribution in BSL significantly differed from those in TAP3 and the random control condition, where distribution followed a more linear shape (*p* < 0.05). During TAP1 and TAP2, the frequency distribution of reaction times retained a sigmoidal profile and differed from the random control (*p* < 0.05; see Fig. 2B for a more detailed analysis). These results are supported by analysis of the IQR (25–75% range; see box plot in Fig. 2B), as a one-way RM ANOVA revealed a significant effect of TAP condition on IQR (F_4,36_ = 43.769, *p* < 0.001). Post hoc testing showed that the IQR of the TAP3 and in the random control condition was significantly larger than that in BSL (*p* < 0.03). Furthermore, the IQR of TAP1, 2 and 3 was significantly smaller than that of the random control condition (*p* = 0.01; Fig. 2B).

### Electrophysiology

As the number of correct pokes decreased with increasing difficulty across the TAP conditions, the number of corresponding ERP trials available also decreased. After baseline correction for the target tone, the BSL condition comprised 519 trials, TAP1 comprised 360 trials, TAP2 comprised 288 trials, and TAP3 comprised 140 trials. In the grand-average ERPs, across all TAP conditions an early ERP component (50–190 ms) could be differentiated from a late component (350–650 ms; Fig. 2C). For comparability, data from the standard FA condition were also included. A permutation ANOVA revealed two significant time segments. One segment occurred in the early ERP component (65–170 ms, F_max = 18.2, *p* < 0.001), whereas the other was located in the late ERP component (374–650 ms, F_max = 13.2, *p* < 0.001; Fig. 2C). Post hoc comparisons with FDR correction showed that amplitudes in early ERP component were higher in BSL, TAP1, and TAP2 than in TAP3 and in the standard FA condition. For the late ERP component, amplitudes in the TAP1 condition were significantly higher than in BSL, TAP3, and the standard FA condition. The standard FA amplitude was reduced compared to BSL, TAP1, and TAP2. No significant differences were found between BSL and TAP2, between TAP1 and TAP2, or between TAP3 and the standard FA condition. Effect sizes (Cohen’s d) were generally in the small to moderate range across significant comparisons (*p* < 0.05; Fig. 2C). More detailed analysis of peak amplitudes (Fig. 2D) revealed significant effects of TAP condition on both the early (F_4,2116_ = 24.462, *p* < 0.001) and late ERP component (F_4,2116_ = 9.292, *p* < 0.001). Post hoc tests showed that early amplitudes were progressively reduced in TAP3 and the standard FA condition (all *p* < 0.01), while peak amplitudes of TAP1, TAP2, and BSL did not differ. For the late component (350–650 ms), the ERP amplitude was enhanced during the TAP2 condition compared to BSL (*p* = 0.022), but decreased during TAP3 and the standard FA condition (*p* < 0.01) without differing from BSL. Inspection of the corresponding grand averages without offline filtering and evaluation of the expected temporal response of the acquisition hardware high-pass filter showed that the observed late ERP component did not match the latency and morphology expected for a filter-induced response.

**Fig. 3:**
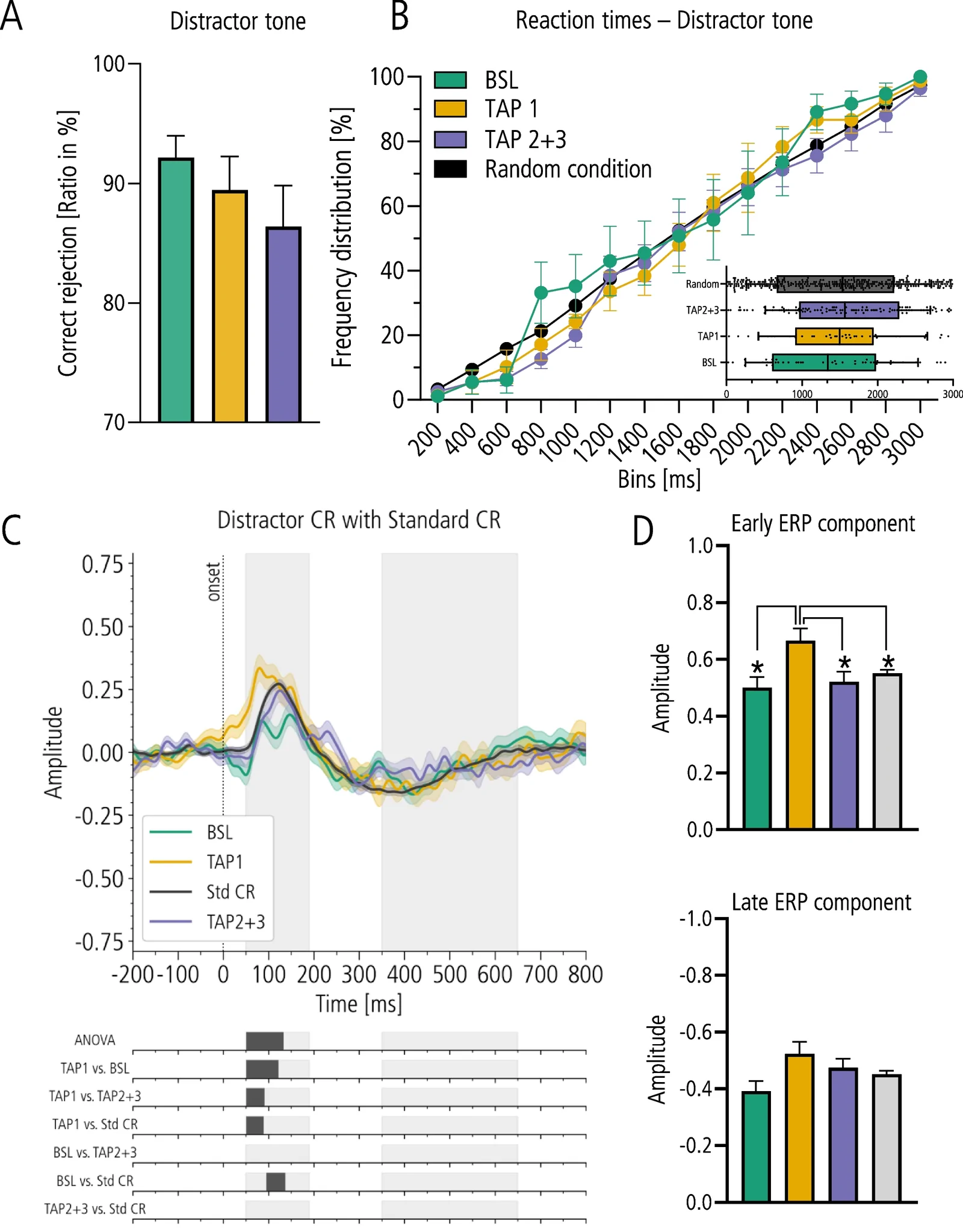
Behavioral and electrophysiology results of the distractor event. Green BSL, yellow TAP1, purple TAP2+3. Significant differences (*p* < 0.05) are visualized by asterisk signs (*). Ratio of correct rejection to the distractor tone. Data are shown as mean + SEM (**A**). Frequency distribution of reaction times for all experimental conditions and the simulated random control condition, as well as box plots (**B**). Grand-averaged ERPs (mean ± SEM) for all conditions, with standard CR condition, aligned to stimulus onset. Gray shaded bands indicate the predefined analysis windows for the early (50–190 ms) and late (350–650 ms) components. Permutation ANOVA shows time segments with significant effects and post hoc comparison (black bars, **C**). Quantitative ERP amplitudes are shown as bar plots summarizing early (top) and late (bottom) amplitudes (mean + SEM), significant differences are visualized by asterisk signs (*p < 0.05; **D**). *BSL* baseline; *CR* correct rejection; *ERP* event-related potential; *Std* standard; *SEM* standard error of the mean; *TAP* Tone Approach Paradigm

The ratio of correct rejections of the distractor tone did not differ between BSL and TAP conditions (Fig. 3A). Furthermore, reaction time analysis of FA to the distractor tone did not differ between conditions (Fig. 3B). For the grand average ERP analysis, the permutation ANOVA revealed a significant segment between 50–132 ms (F_max = 6.5; *p* < 0.1). Post hoc comparisons with FDR correction showed significant segments for the TAP1 condition (all *p* < 0.05, Cohen’s d < 0.26). Additionally, a significant segment was found for comparison between BSL and standard CR in the early component (*p* < 0.05, Cohen’s d: 0.202). Peak amplitude comparison revealed a significant effect on the early amplitude (one-way ANOVA, H_3_ = 14.747, *p* = 0.002), with TAP1 showing a significantly higher amplitude than BSL, TAP2+3, and the standard CR condition (*p* < 0.05). No significant differences were observed for the late ERP amplitude (Fig. 3C/D).

Target hit and target miss trials were only evaluated qualitatively, as miss trials were rare, particularly in BSL and TAP1, and therefore associated with high variance. Especially for the late ERP component in the BSL, TAP1, and TAP2 conditions, target miss trials were less pronounced than target hits. In TAP3, there was almost no difference between target hit and target miss ERPs (Supplementary Fig. S2).

### Discussion

This study examined how the progressive approximation of tone frequencies affects attention, decision-making, and neural processing in a three‑class auditory oddball paradigm. Across TAP conditions, we progressively decreased the frequency contrast between the target and the distractor tone toward the standard frequency and evaluated effects on behavior and mPFC LFP recordings, focusing on early and late ERP components.

For the BSL condition, behavioral performance was high, with correct responses above 80%. The frequency distribution of the reaction times had a sigmoidal shape, indicating precise responding to the target. The ERP showed pronounced peaks in the early and late components, consistent with the patterns previously reported by our group (Decker et al., 2025a) and with similar response patterns described in human studies (Beck et al., 2020; Polich, 2007; Wronka et al., 2008).

In the “low difficulty” TAP1 condition, where the frequency of the target was reduced by 500 Hz, behavior remained comparable to BSL. The hit rate was still above 80% and the reaction time distribution had a sigmoidal shape, indicating preserved temporal clustering of responses to the modified target, supporting the view that responding in TAP1 was still temporally well-structured and stimulus-related. The criterion value close to zero at low difficulty also suggested little response bias and was consistent with accurate target detection with minimal bias under these conditions. However, while the early ERP component amplitude was comparable to that of the BSL condition, the late component was significantly enhanced. In human oddball studies, enhanced late ERP amplitudes have often been linked to increased cognitive processing of sensory information and to the effort required to classify the target stimulus (Kutas et al., 1977; Magliero et al., 1984). The enhanced ERPs extracted during the “low difficulty” TAP1 condition are therefore consistent with an increase in processing effort for target categorization.

In the “moderate difficulty” TAP2 condition, behavioral performance declined significantly relative to BSL, as reflected by a reduction in hits and d’. The reduction in d′ may primarily reflect reduced target detectability rather than changes in distractor processing, as FAs to distractors remained largely unchanged across conditions. Moreover, criterion was only slightly increased, suggesting that stimulus discrimination was still largely intact and responses remained stimulus-driven. When rats responded to the target tones, reaction times still showed high precision, suggesting that target misses may instead have reflected false categorization of the modified target frequency. Consistent with this, the reaction time distribution in TAP2 also retained a sigmoidal profile, indicating that responding was still temporally structured despite reduced performance. Although correct hits were reduced in TAP2, the amplitudes of the early and late ERP components for correct hits were similar to those observed in TAP1, suggesting that target evaluation and decision-related processing may have remained preserved on correct trials. This pattern is consistent with observations from difficult tasks in human oddball studies (Kok, 2001; Polich, 2007).

For the “high difficulty” TAP3 condition, the hit rate still remained above chance level, but dropped significantly below 40%. The cumulative reaction time distribution lost its pronounced sigmoidal shape and became flatter and more linear, indicating that the rats had substantial difficulty identifying the heavily modified target frequency in this condition. Nevertheless, it was still not fully random, as indicated by a clear “deadtime” after stimulus onset, suggesting that stimulus discrimination was still present but clearly disturbed. The cumulative reaction time distribution in TAP3 was significantly different from BSL but did not differ from that in a simulated random control condition. Signal detection measures also supported this interpretation. While in TAP1 and TAP2 conditions, criterion values remained similar to BSL, consistent with little overall response bias under conditions in which responding was still largely stimulus-driven, criterion in TAP3 shifted markedly toward positive values. Although a positive criterion is classically interpreted as a conservative response bias, this explanation alone seems insufficient to account for the present data. In the present task, in addition to moving to the back hole in vain, FAs were followed by a brief 1000-ms timeout and thus carried a small price. The increase in criterion in TAP3 may therefore partly reflect an attempt to avoid such FAs. However, within signal detection theory, shifts in criterion may also reflect changes in decision strategy under uncertainty and altered sensory evidence, rather than cautious responding alone (Rahnev, 2021; Won Bang & Rahnev, 2017). Important, the number of FA across conditions was similar in all conditions, and therefore the increase in criterion primarily depended on altered behavior following the target stimulus in TAP3. Together with the reduced d’, the more linear reaction time distribution, and the weak separation between hit and miss ERPs in the TAP3 condition, the positive criterion suggests that target-driven responding was impaired and that at least part of the remaining hits may have reflected less tightly stimulus-locked responding, rather than solely a shift toward a more conservative response bias.

The behavioral findings were also reflected in the ERPs recorded in the TAP3 condition. Both the early and late components decreased in amplitude and did not differ from those of the ERPs in the standard FA condition, which the rats had learned to ignore during training (Decker et al., 2025a). For the late ERP amplitude in the high difficulty TAP3 condition, two complementary mechanisms may account for this reduction. First, increasing task difficulty likely depleted attentional resources, leaving less capacity for the stimulus evaluation processes reflected in the late component (Isreal et al., 1980; Wickens et al., 1983). Second, when the target frequency in TAP3 moved closer to the standard frequency, it may no longer have been perceived as a distinct or rare event. In such cases, the target would lose its behavioral relevance and may be processed more like a standard, thereby diminishing the context-updating response typically associated with target detection. In addition, oddball paradigms typically show reduced early ERP amplitudes for frequent standard tones, a phenomenon commonly referred to as stimulus-specific adaptation, which has been described in barn owls and humans (Gutfreund, 2012; Rosburg & Mager, 2021). The smaller early ERP amplitude observed in the TAP3 condition may therefore partly reflect the closer frequency match between target and standard tones, which could promote adaptation- or habituation-like attenuation of neural responses and reduced behavioral relevance.

Across TAP conditions, ERP amplitudes, particularly the late component, showed an inverted U-shaped relationship with task difficulty, increasing at low and moderate difficulty levels (TAP1 and TAP2) and decreasing again at the highest difficulty level (TAP3). The late component therefore likely reflects task-related processing of behaviorally relevant stimuli, whereas the marked decline at the highest difficulty level in TAP3 coincided with behavioral deterioration, less tightly stimulus-locked reaction times, and a strong shift in criterion. This pattern suggests that the late component is most strongly engaged at intermediate levels of task difficulty, but may diminish when task difficulty becomes too high to support reliable stimulus-driven behavior.

Qualitative comparison of hits and misses in BSL, TAP1, and TAP2 showed clear differences at the ERP level, with enhanced late components on correct trials but markedly smaller amplitudes on incorrect trials, suggesting that the “decision stage” may involve the mPFC. This pattern is consistent with our previous findings showing differences between target hits and target misses (Decker et al. 2025a). In TAP3, however, this separation was almost lost, consistent with the view that hits in TAP3 may have become less tightly coupled to the stimulus and occurred more sporadically.

Together, the behavioral measures “hit rate” and “reaction time distribution,” as well as the ERP analysis in the TAP paradigm, allow us to distinguish preserved target-locked responding and conditions with reduced temporal response clustering and impaired target discrimination. Together, these findings suggest that reduced performance in the high-difficulty condition is not simply the result of a general decline in performance, but rather reflects a reduction in target-driven responding. However, although the random control condition was used as a descriptive reference for sporadic poking independent of stimulus timing, it does not represent the only possible model of random responding. Other response patterns are conceivable. For example, studies using rat waiting paradigms suggests that broadly distributed response times can also arise from variable waiting-related processes and decision dynamics, rather than solely from a simple uniform process (Murakami et al., 2014). Therefore, the uniformly distributed random control condition was used here as an intuitive benchmark for reduced temporal clustering, not as a definitive model of all random or unspecific poking behavior. Moreover, the observation of an increased late ERP amplitude under the “low-” and “moderate-difficulty” conditions (TAP1 and TAP2), compared with the BSL condition, may reflect increased task-related neural processing involved in evaluating and categorizing targets under challenging, yet still achievable, target tone manipulation tasks. This profile is compatible with resource‑based models of task difficulty effects on late ERP components (Isreal et al., 1980; Kok, 2001; Wickens et al., 1983).

Consistent with Quintela-Vega et al. (2023), the present findings show that freely moving rats can detect deviant auditory stimuli in a classical active oddball paradigm and that performance depends on stimulus discriminability. However, the paradigms differ substantially: Quintela-Vega et al. used a two-class oddball design and assessed rat performance mainly by increasing the deviant-standard frequency contrast. In comparison, the present TAP paradigm progressively reduces deviant discriminability by decreasing the frequency contrast between the target and the standard tone and between the distractor and the standard tone within a three-class design. Although in their “many-deviant task” numerous different deviant frequencies were used, only one of these frequencies lay between the standard and the target, i.e., it resembled one of the frequencies implemented in our TAP paradigm (Quintela-Vega et al., 2023). Similar to our results, rat performance was markedly impaired in this particular context. However, this comparison should be made with caution, as Quintela-Vega’s group analyzed rat behavior within a broader many-deviant framework rather than in terms of progressively reduced discriminability of target and distractor tones from the standard tone within a three-class design. In addition, some deviants were presented below the standard frequency, but these are not directly comparable to our distractors, as they still served as response-relevant deviants rather than as rare, behaviorally irrelevant stimuli. Thus, while both studies support the general conclusion that performance improves as the relevant stimulus becomes more distinct from the standard, our approach specifically addresses how behavior and frontal ERP components change as target discriminability deteriorates.

With respect to the distractor tone, behavioral performance did not change across TAP sessions, but the early ERP amplitude in the TAP1 condition was enhanced. The early amplitude is typically associated with automatic or obligatory processing of incoming stimuli. As the rats were extensively trained to respond to the target tone, while ignoring standard and distractor tones, they likely categorized both the standard and distractor tones as “non-target” stimuli rather than as frequent standards or rare novel distractor (Decker et al., 2025a, b). This differs from human settings, where the oddball paradigm is typically performed with high precision soon after instruction. In this context, the standard tone is usually passively ignored, whereas the rare distractor requires active response suppression, resulting in an enhanced ERP amplitude for the distractor compared with the standard stimuli (Beck et al., 2018; Debener et al., 2002). In TAP1, introducing a new distractor frequency still enhanced the early amplitude, probably because this modulated tone was perceived as a “new” oddball stimulus. However, in the most difficult conditions, where the distractor and standard frequencies were highly similar, this effect was no longer evident. This was likely because the rats could no longer reliably discriminate between the two tones, meaning that the distractor was functionally more similar to the standard.

A limitation of the present TAP implementation is that the frequency distance between target and standard and between distractor and standard was based on fixed 500 Hz steps rather than logarithmic or octave-scaled steps. As a result, the psychoacoustic distance between tones was not constant across conditions, and the spacing of target and distractor around the standard was not perfectly symmetric in octave terms across all stages. Future implementations may adopt more logarithmic frequency steps or octave-based scaling to enforce symmetric frequency separations around the standard.

In addition, the target was consistently higher and the distractor was consistently lower than the standard tone. Our approach provides a controlled and scalable way to reduce discriminability relative to the constant 3000 Hz standard, it also introduces a potential confound between “task difficulty” and “tone frequency” of the paradigm, which may evoke different neural responses independent of task demands. This potential sensory confound should therefore be considered when interpreting ERP differences across TAP conditions, as the present design does not fully dissociate effects of increasing task difficulty from possible effects of absolute tone frequency on the recorded responses. Recent work, however, showed that behavior and prefrontal ERP did not differ when either 1500 Hz or 5000 Hz was used as the target frequency, suggesting that the neural correlate of target detection and categorization during testing in the three-class oddball paradigm are largely independent of absolute target frequency (Akbarzadeh et al., 2025). In addition, deviance-related ERP-like responses recorded from the posterior parietal cortex of rats also do not differ under conditions of high and low tone frequency, arguing against a purely frequency-driven account of such effects (Imada et al., 2013).

Animal models are widely used in neuroscience, but they do not capture all aspects of human cognition and brain organization (Zhao & Bhattacharyya, 2018). Although rodents and humans share general principles of neural organization, important differences remain in brain size, relative regional volumes, and connectivity patterns (Schaeffer et al., 2020). These differences are relevant to interpreting the spatial origin of electrophysiological signals. LFPs are often considered relatively local, typically reflecting activity within about 200–400 µm of the electrode tip (Xu et al., 2022), but they can also reflect activity extending across several millimeters (Kajikawa & Schroeder, 2011). Therefore, despite the fact that the mPFC and the auditory cortex in rats are separated by at least 5 mm, the recorded mPFC potentials may represent a mixture of sensory responses and the more cognitive processes typically associated with the frontal cortex. This limitation should be considered when interpreting the mPFC signals in the present study. Moreover, the circuitry underlying auditory information processing is highly complex and involves multiple projections from the auditory cortex to higher-order prefrontal cortical regions that may mediate rapid and dynamic changes in response properties (Fritz et al., 2010; Hockley et al., 2025; Hockley & Malmierca, 2024; Winkowski et al., 2013).

## Conclusions

The TAP paradigm extends the auditory three-class oddball paradigm framework by creating a scalable cognitive workload model using systematically manipulated auditory stimuli. Integrating behavioral and electrophysiological metrics enables a comprehensive assessment of cognitive capacity, its limitations, and the underlying neurobiological mechanisms. In addition, we introduced the frequency distribution of reaction times as a valuable behavioral parameter. This approach could be valuable for future research exploring attentional dysfunction in models of neuropsychiatric and neurodegenerative diseases. Beyond the specific findings reported here, TAP provides a flexible translational framework for future work investigating attentional dysfunction and stimulus evaluation in preclinical models.

## Supplementary Information

Below is the link to the electronic supplementary material.

Supplementary Fig. S1 Behavior signal detection measures across all TAP conditions. D-prime (d’) is shown in the upper panels and criterion in the lower panels. Comparisons between target and distractor are shown on the left, and comparisons between target and standard on the right. Bar graphs show mean + SEM, and dots represent individual animals. Significant differences are marked by asterisks (*p < 0.05). BSL baseline; SEM standard error of the mean; TAP Tone Approach Paradigm. (PDF 176 KB)

Supplementary Fig. S2 Comparison of target hit and target miss ERPs across all TAP conditions. Target hit trials are shown in red, and target miss trials are shown in orange. Grand-average ERPs are presented as mean ± SEM. BSL baseline; ERP event-related potential; SEM standard error of the mean; T target; TAP Tone Approach Paradigm. (PDF 462 KB)

## Data Availability

The datasets generated and analyzed during the current study, are available from the corresponding author upon reasonable request.

## Code Availability

The analysis code used in the current study is available from the corresponding author upon reasonable request.
