# Supplementary material for: Modulating auditory attention and stimulus evaluation in the medial prefrontal cortex: A translational tone approach oddball paradigm in rats
Source: Cogn Affect Behav Neurosci. 2026 Jul 7;26(5):2105–17. doi: 10.3758/s13415-026-01473-7 (PMC13614979; doi:10.3758/s13415-026-01473-7)
Supplement: Supplementary file 1 [file 13415_2026_1473_MOESM1_ESM.pdf]

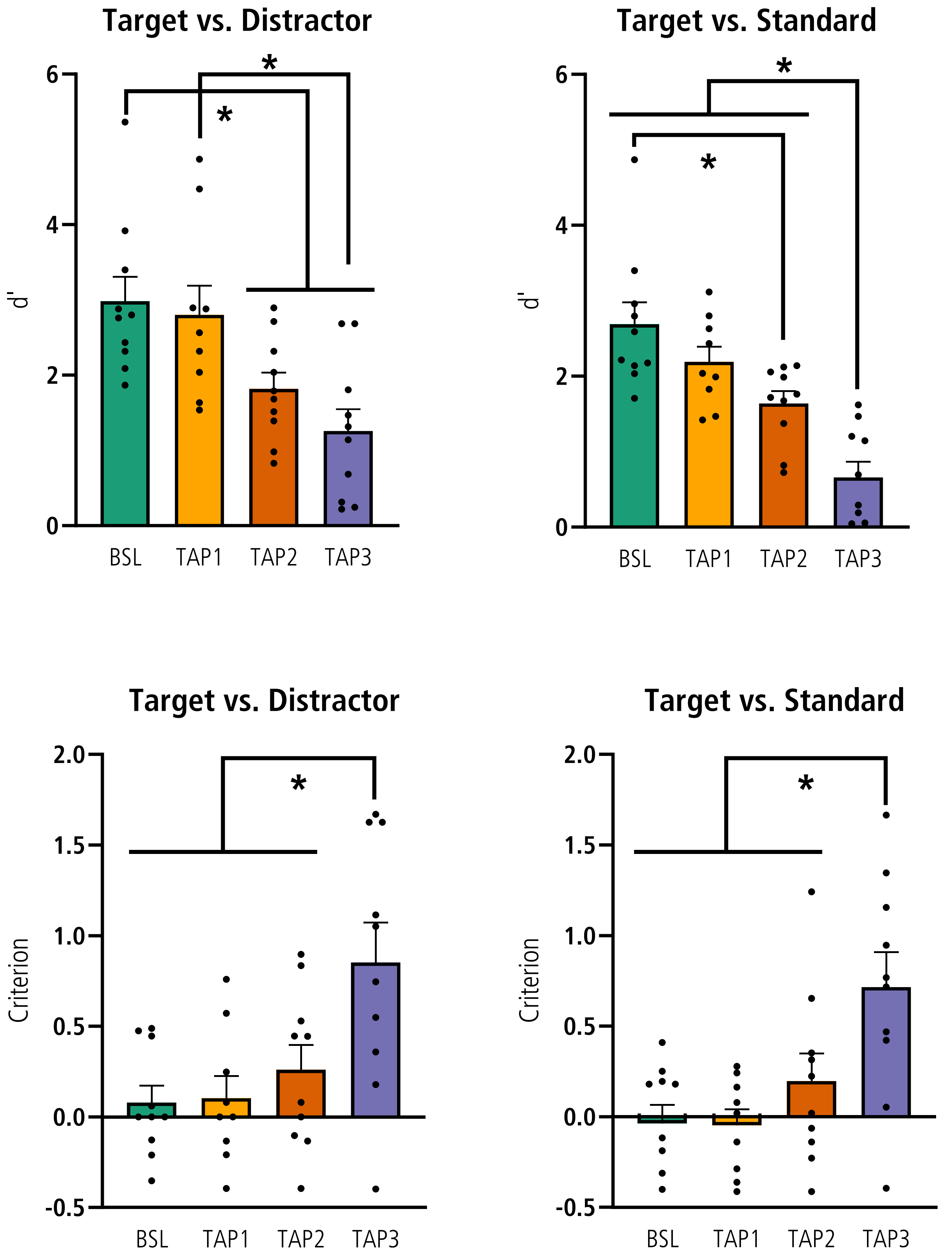

Target vs. Distractor
Target vs. Standard
d'
Criterion
BSL
TAP1
TAP2
TAP3
*
