# Supplementary material for: Modulating auditory attention and stimulus evaluation in the medial prefrontal cortex: A translational tone approach oddball paradigm in rats
Source: Cogn Affect Behav Neurosci. 2026 Jul 7;26(5):2105–17. doi: 10.3758/s13415-026-01473-7 (PMC13614979; doi:10.3758/s13415-026-01473-7)
Supplement: Supplementary file 2 [file 13415_2026_1473_MOESM2_ESM.pdf]

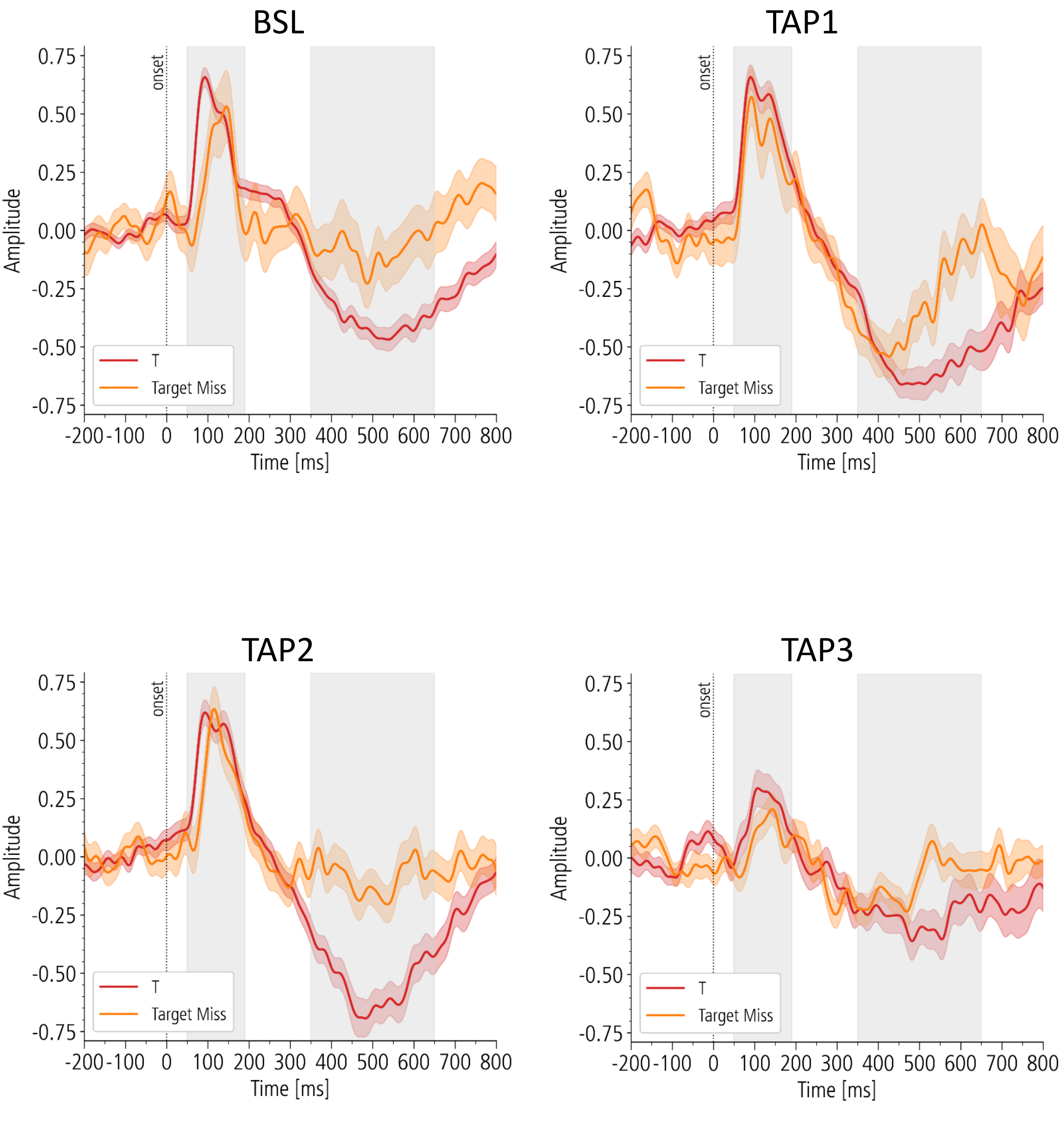

BSL
TAP1
TAP2
TAP3
onset
Amplitude
Time [ms]
T
Target Miss
0.75
0.50
0.25
0.00
-0.25
-0.50
-0.75
-200
-100
0
100
200
300
400
500
600
700
800
